# Botanical drug preparations for alleviating hair loss in menopausal women: a global ethnopharmacological mini-review

**DOI:** 10.3389/fphar.2025.1725691

**Published:** 2025-11-26

**Authors:** Zhuo Huang, Wen-Jie Zhao, Yong-Jie Gao, Zhi-Rong Huang, Qing-Rui Huang, Sui-Zhen Cai, Ming-Hui Bi

**Affiliations:** 1 Xiamen Hospital of Traditional Chinese Medicine, Xiamen, Fujian, China; 2 The Second People’s Hospital Affiliated to Fujian University of Traditional Chinese Medicine, Fuzhou, Fujian, China

**Keywords:** menopausal hair loss, ethnopharmacology, traditional remedies, botanical drugs, phytoestrogens, hair follicle regeneration, women’s empowerment, global health perspectives

## Abstract

Menopause is a natural physiological transition affecting over one billion women globally. It often brings distressing symptoms, including hair loss, which impacts up to 52% of postmenopausal women due to estrogen decline, increased androgen sensitivity, and oxidative stress. Conventional treatments like minoxidil provide limited efficacy and may cause side effects, underscoring the need for accessible, culturally attuned alternatives. This mini-review explores ethnopharmacological approaches that empower women through traditional remedies targeting multi-mechanistic pathways, supported by emerging clinical data. We conducted a systematic literature search across databases (PubMed, Scopus, Web of Science), focusing on studies from 2015 to 2025 involving botanical interventions. We prioritized randomized controlled trials *in vitro* studies, and animal models. Key remedies include saw palmetto (*Serenoa repens*), which inhibits 5α-reductase to reduce dihydrotestosterone by approximately 30%–40%; rosemary oil (*Salvia rosmarinus* Spenn.), which promotes scalp microcirculation similar to minoxidil; and ginseng (*Panax ginseng* C.A.Mey.), which enhances follicle proliferation via ginsenosides. Emerging evidence from 2024 to 2025 reviews highlights multi-target mechanisms in plant extracts, including phytoestrogenic and anti-inflammatory effects. Nutraceuticals have demonstrated improved hair density in menopausal cohorts. These low-cost, community-rooted therapies foster women’s autonomy and cultural resilience. This mini-review is not comprehensive; it highlights key challenges in current research, such as limited menopausal-specific evidence and standardization gaps. It advocates for future priorities like interdisciplinary trials integrating ethnobotany with modern pharmacology to bridge global health disparities. This aligns with the special issue’s vision of empowering women through sustainable, nature-based solutions.

## 1 Introduction

Menopause marks a pivotal physiological transition in women’s lives. It is characterized by the cessation of ovarian function and a decline in estrogen levels. By 2030, it will affect over 1.2 billion women worldwide (Spector et al., 2024). This natural process typically occurs between ages 45 and 55, bringing a cascade of symptoms beyond well-known vasomotor disturbances like hot flashes and night sweats, which profoundly impact quality of life (Andrews et al., 2024). Among them, hair loss—often as female pattern hair loss (FPHL) or diffuse thinning—is a subtle yet pervasive challenge that erodes women’s self-esteem, social confidence, and emotional wellbeing (Young Moss et al., 2025). Studies show that up to 52% of postmenopausal women experience noticeable hair thinning, underscoring its status as an underrecognized menopausal burden (Shannon et al., 2015; Chaikittisilpa et al., 2022; Ho et al., 2023). Prevalence varies globally due to genetics, diet, and environment, and the symptom can intensify psychological distress, elevating risks of depression and anxiety while compounding socioeconomic disparities in women’s health equity (Malta and Corso, 2025).

Conventional management strategies, such as topical minoxidil or hormone replacement therapy (HRT), offer partial relief but face limitations, including Side effects like scalp irritation and cardiovascular risks. Long-term adherence is often poor, especially in diverse global contexts (Dakkak et al., 2024). This gap highlights the potential of ethnopharmacology, rooted in traditional knowledge systems of various ethnic or cultural groups, to provide accessible, culturally resonant alternatives that empower women to reclaim agency over their health (Shin et al., 2020; Kesika et al., 2023; Elnady et al., 2025). Traditional remedies range from Ayurvedic bhringraj oils in India to Mediterranean rosemary infusions and African shea butter formulations. They have long addressed hair vitality through multi-target mechanisms, such as phytoestrogen modulation and anti-inflammatory action (Ezekwe et al., 2020; Ahmed et al., 2025). While promising, botanical remedies do not achieve complete hair regrowth, as indicated by current clinical evidence (Elnady et al., 2025). Recent reviews emphasize HRT’s role in symptom mitigation; however, few integrate global ethnobotanical perspectives, overlooking how community-sourced therapies foster resilience and autonomy in underserved populations (Nappi et al., 2021).

This mini-review addresses these gaps by synthesizing evidence on ethnopharmacological interventions for menopausal hair loss, using a global health lens that prioritizes women’s empowerment. It draws from diverse traditions, such as Asian ginseng extracts, European nettle root and American saw palmetto, elucidating mechanisms, efficacy, and safety profiles. It advocates for equitable research to bridge cultural and economic divides. This work aligns with the special issue’s focus on “Global Health Perspectives on Empowering Women.” Demonstrating how revitalizing ancestral remedies can transform menopause into a phase of holistic flourishing.

Given the breadth of global ethnopharmacology, this mini-review does not aim for exhaustive coverage. It selects representative botanical drugs to underscore key research challenges, including a paucity of large-scale, menopausal-focused trials. It outlines future needs, such as standardized formulations and equitable community-engaged studies, aligning with the special issue’s focus on empowering women through sustainable solutions.

## 2 Methodology

This mini-review adopts a systematic approach, prioritizing narrative synthesis of select studies to highlight evidence gaps and research priorities. It does not include a full meta-analysis due to heterogeneity. We searched electronic databases, including PubMed, Scopus, and Web of Science. The search covered studies published between January 2015 and November 2025. The strategy used keywords and MeSH terms such as “menopausal hair loss,” “female pattern hair loss,” “ethnopharmacology,” “traditional medicine,” “botanical drug remedies,” “phytoestrogens,” “hair follicle,” and “women’s health.” We combined them with Boolean operators (e.g., AND, OR). We also searched gray literature sources like Google Scholar and ethnobotanical databases (e.g., NAPRALERT) for non-indexed publications, and hand-searched reference lists of included articles and relevant reviews for additional studies.

**FIGURE 1:**
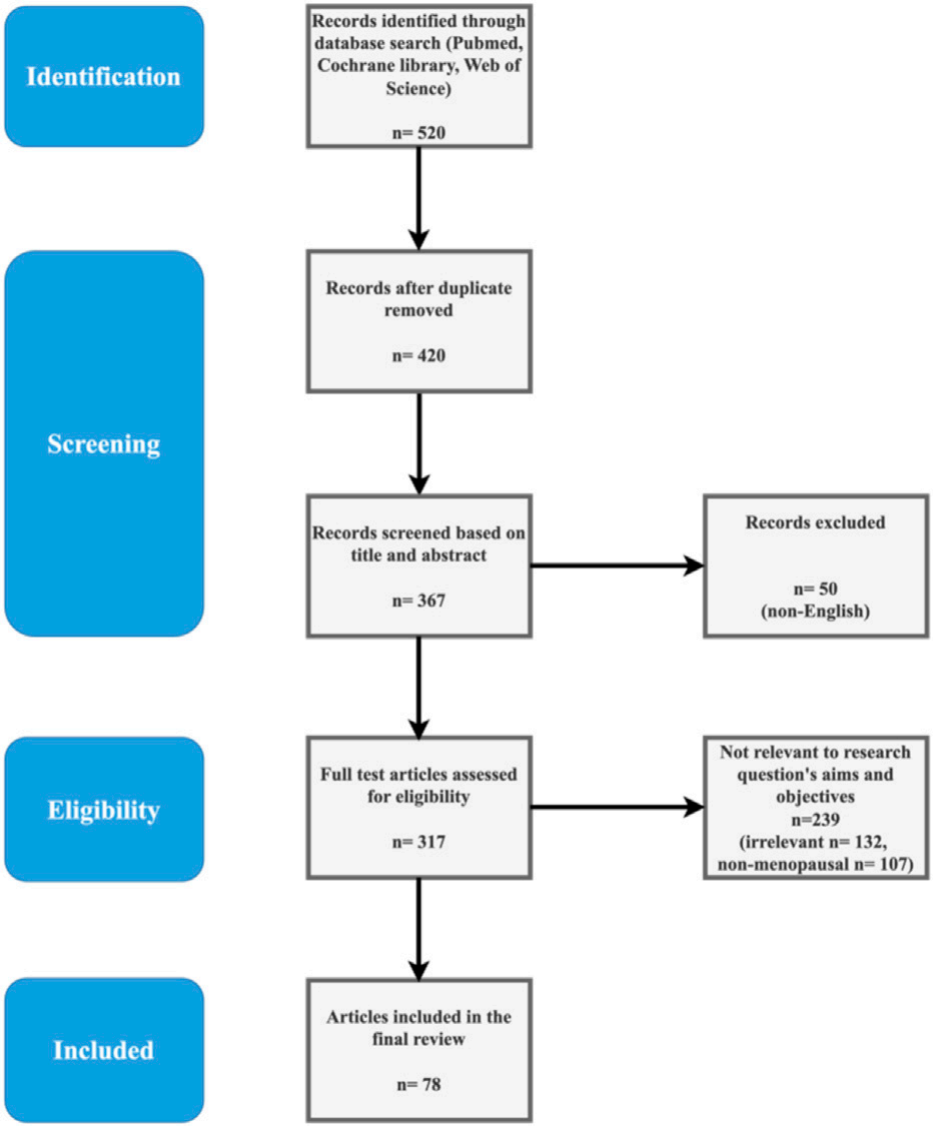
PRISMA-inspired flow diagram for literature selection.

Inclusion criteria focused on studies evaluating plant-based or traditional remedies for hair loss in menopausal or postmenopausal women, including RCTs, *in vitro* experiments, animal models, observational studies, and ethnopharmacological surveys. We prioritized human trials but included preclinical data for mechanistic insights. Exclusion criteria included non-English articles, studies on non-menopausal hair loss (e.g., chemotherapy-induced), synthetic pharmaceuticals without botanical components, and low-quality case reports. Two reviewers independently screened titles and abstracts, assessed full texts for eligibility, and resolved discrepancies through discussion. The selection process is illustrated in Figure 1, a PRISMA-style flow diagram showing records identified (n = 520), screened (n = 420 after duplicates), excluded (n = 367, e.g., non-English n = 50, irrelevant n = 132, non-menopausal n = 107), and included (n = 78).

Data extraction included key details on remedies (e.g., plant species, active metabolites), mechanisms, efficacy outcomes (e.g., hair density, shedding rates), safety profiles, and cultural contexts. We appraised evidence quality using tools like GRADE for clinical trials and STROBE for observational studies. Formal meta-analysis was not performed due to heterogeneity. Synthesis was narrative, grouped by geographic regions and themes, emphasizing global perspectives on women’s empowerment. A total of 78 studies were included, providing a comprehensive overview while highlighting research gaps.

## 3 Pathophysiology and epidemiology of menopausal hair loss

Menopausal hair loss primarily manifests as female pattern hair loss (FPHL) or telogen effluvium, arising from intricate hormonal and physiological changes during the menopausal transition (Fabbrocini et al., 2018; Gupta et al., 2025). The pathophysiology is driven by a sharp decline in estrogen levels, which disrupts the hair growth cycle by shortening the anagen (growth) phase and extending the telogen (resting/shedding) phase, leading to increased hair shedding and reduced density (Gupta et al., 2025; Rinaldi et al., 2023). Relative androgen dominance also plays a role, exacerbated by the conversion of testosterone to DHT via 5α-reductase, promoting follicular miniaturization. Terminal hairs transform into finer vellus hairs, especially in androgen-sensitive scalp regions like the frontal and vertex areas (Gupta et al., 2025; Grymowicz et al., 2020; Perez et al., 2024). Oxidative stress and inflammation, intensified by estrogen withdrawal, impair scalp microcirculation and nutrient delivery to hair follicles, contributing to atrophy and altered hair characteristics, such as decreased caliber and pigmentation (Rinaldi et al., 2023). Genetic predisposition (e.g., polymorphisms in androgen receptor genes) and metabolic changes like insulin resistance amplify these effects, distinguishing menopausal hair loss from age-related thinning (Rinaldi et al., 2023). Comorbidities like thyroid dysfunction or iron deficiency, common in perimenopause, can worsen the condition (Hussein et al., 2023; Lin et al., 2023).

Epidemiologically, menopausal hair loss affects a substantial proportion of women globally, with prevalence increasing with age and hormonal status. Studies estimate that clinically detectable FPHL emerges in 12% of women by age 29, rising to 25% by age 49, 41% by age 69, and exceeding 50% in those over 70, highlighting a strong association with postmenopausal years (Fabbrocini et al., 2018). In postmenopausal cohorts, prevalence reaches up to 52.2%, with a mean age of onset around 58 years (Chaikittisilpa et al., 2022; Ho et al., 2023). Regional variations exist: In Western populations (e.g., Europe and North America), rates are 40%–50% among perimenopausal and postmenopausal women, influenced by diagnostic awareness and genetic factors like Caucasian ethnicity (Shannon et al., 2015). Asian populations show lower prevalence, around 6%–12% across ages, with rates as low as 1.3% in younger groups but increasing postmenopausally, possibly due to protective dietary elements like phytoestrogens (Besong et al., 2024). In low-resource settings like parts of Africa and Latin America, underreporting likely masks higher burdens, with nutritional deficiencies and chronic stress potentially elevating risks to 40%–60%, exacerbating global health inequities (Rinaldi et al., 2023; Goluch-Koniuszy, 2016). Overall, FPHL accounts for nearly 49% of female alopecia cases over a lifetime, with socioeconomic factors like access to care widening disparities.

These insights underscore the need for targeted interventions, especially in diverse global contexts where conventional therapies fall short. Ethnopharmacological approaches, drawing from traditional knowledge, offer holistic ways to address these mechanisms, as explored below.

## 4 Ethnopharmacological approaches: a global overview

Ethnopharmacological traditions worldwide offer diverse, multi-target botanical drugs for menopausal hair loss, rooted in cultural practices and supported by primary ethnobotanical references. This section synthesizes representative remedies by region, emphasizing traditional contexts and evidence. It highlights how these approaches empower women through accessible, community-based solutions (see Table 1 for regional summaries; Table 2 for detailed pharmacological data, including mechanisms, efficacy, and limitations).

**TABLE 1:** Key ethnopharmacological remedies for menopausal hair loss by region.

Region	Family	Scientific name (Author)	Common name	Active metabolites	Mechanisms/Evidence	References
Asia	Apiaceae	*Angelica sinensis* (Oliv.) Diels [Apiaceae; Angelicae sinensis radix]	Dang Gui	Polysaccharides, ferulic acid	Tonifies blood, promotes circulation	Song et al. (2021)
Asia	Araliaceae	*Panax ginseng* C.A.Mey. [Araliaceae; Ginseng radix et rhizoma]	Ginseng	Ginsenosides	Enhances follicle proliferation	Zaid et al., 2017; Choi (2018)
Asia	Asteraceae	*Eclipta prostrata* (L.) L. [Asteraceae; Ecliptae herba] (syn. *Eclipta alba* (L.) Hassk.)	Bhringraj	Wedelolactone	Hair growth stimulation	Dudhat et al. (2024)
Asia	Fabaceae	*Glycine max* (L.) Merr. [Fabaceae; Sojae semen]	Soy	Isoflavones	Phytoestrogens, DHT inhibition	Luan et al. (2025)
Asia	Fabaceae	*Sophora flavescens* Aiton [Fabaceae; Sophorae flavescentis radix]	Sophora root	Matrine, oxymatrine	IGF-1 induction, anagen promotion	Roh et al. (2002), da Cruz et al. (2022)
Asia	Oleaceae	*Ligustrum lucidum* W.T.Aiton [Oleaceae; Ligustri lucidi fructus]	Ligustrum	Oleanolic acid	Reduces oxidative stress	Choi, 2018; Park et al. (2015)
Asia	Orobanchaceae	*Rehmannia glutinosa* (Gaertn.) DC. [Orobanchaceae; Rehmanniae radix]	Rehmannia	Catalpol, iridoids	HSC quiescence, anti-senescence	Song et al. (2021)
Asia	Paeoniaceae	*Paeonia lactiflora* Pall. [Paeoniaceae; Paeoniae radix alba]	Paeonia	Paeoniflorin	Steroid hormone suppression	Cui et al. (2025)
Asia	Phyllanthaceae	*Phyllanthus emblica* L. [Phyllanthaceae; Phyllanthi fructus]	Amla	Vitamin C	Antioxidant, proliferation	Yamakami et al. (2019), Majeed et al. (2020)
Asia	Polygonaceae	*Reynoutria multiflora* (Thunb.) Moldenke [Polygonaceae; Polygoni multiflori radix] (syn. *Polygonum multiflorum* Thunb.)	He Shou Wu	Stilbenes	Anagen prolongation	Choi, 2018; Park et al. (2015)
Europe/Mediterranean	Annonaceae	*Cananga odorata* (Lam.) Hook.f. and Thomson [Annonaceae; Canangae odoratae flos]	Ylang-ylang	Essential oils	Regulates sebum, stimulates growth, relieves inflammation	Pareek et al. (2023)
Europe/Mediterranean	Equisetaceae	*Equisetum arvense* L. [Equisetaceae; Equiseti herba]	Horsetail	Silica	Strengthens hair, promotes growth via silicon	Broderick et al. (2021)
Europe/Mediterranean	Lamiaceae	*Lavandula angustifolia* Mill. [Lamiaceae; Lavandulae flos]	Lavender	Linalool	Reduces stress, anti-inflammatory for scalp	Hawkins et al. (2020), Abelan et al. (2022)
Europe/Mediterranean	Lamiaceae	*Mentha* × *piperita* L. [Lamiaceae; Menthae piperitae folium]	Peppermint	Menthol	Enhances circulation	Ham et al. (2025)
Europe/Mediterranean	Lamiaceae	*Salvia rosmarinus* Spenn. [Lamiaceae; Rosmarini folium]	Rosemary	Carnosic acid	Improves microcirculation	Bin Rubaian et al. (2024)
Europe/Mediterranean	Urticaceae	*Urtica dioica* L. [Urticaceae; Urticae radix]	Nettle	Beta-sitosterol	DHT blocker	Hasannejad-Asl et al. (2025)
Americas	Arecaceae	*Serenoa repens* (W.Bartram) Small [Arecaceae; Serenoae repentis fructus]	Saw palmetto	Fatty acids	DHT inhibitor	Evron et al. (2020)
Americas	Asphodelaceae	*Aloe vera* (L.) Burm.f. [Asphodelaceae; Aloes folii succus]	Aloe vera	Enzymes, vitamins	Hydrates scalp	Abid et al. (2025)
Americas	Rosaceae	*Prunus africana* (Hook.f.) Kalkman [Rosaceae; Pruni africanae cortex]	Pygeum	Beta-sitosterol	Reduces inflammation	Taiti et al. (2024)
Americas	Simmondsiaceae	*Simmondsia chinensis* (Link) C.K.Schneid. [Simmondsiaceae; Simmondsiae chinensis semen]	Jojoba	Wax esters	Balances oil production	Abdalla et al. (2024), Gad et al. (2021)
Africa/Other	Cucurbitaceae	*Cucurbita pepo* L. [Cucurbitaceae; Cucurbitae peponis semen]	Pumpkin seed	Fatty acids, Phytoestrogens	DHT inhibition, promotes anagen growth	Ibrahim et al. (2021)
Africa/Other	Moringaceae	*Moringa oleifera* Lam. [Moringaceae; Moringae folium]	Moringa	Flavonoids, vitamins, isothiocyanates	Antioxidant, anti-alopecia, DHT inhibition	Broderick et al. (2021)
Africa/Other	Oleaceae	*Olea europaea* L. [Oleaceae; Olivae oleum]	Olive	Oleuropein, hydroxytyrosol	Promotes anagen via Wnt/β-catenin, anti-inflammatory	Tong et al. (2015)
Africa/Other	Ranunculaceae	*Nigella sativa* L. [Ranunculaceae; Nigellae sativae semen]	Black seed	Thymoquinone, nigellone	Antioxidant, anti-inflammatory, hair growth promotion	Broderick et al. (2021)
Africa/Other	Sapotaceae	*Vitellaria paradoxa* C.F.Gaertn. [Sapotaceae; Butyrospermi parkii butyrum]	Shea butter	Fatty acids	Anti-inflammatory	Megnanou and Niamke (2015), Isaac et al. (2023)

**TABLE 2:** Detailed pharmacological data for key remedies.

Botanical drug	Extract type/Composition	Study type/Model	Dose/MAC/Duration	Controls	Key findings	Limitations/Gaps	References
*Panax ginseng* C.A.Mey. [Araliaceae; Ginseng radix et rhizoma]	Ethanolic root extract (70%–80%), standardized to 20%–40% ginsenosides (HPLC: Rb1, Rg1, Re)	*In vivo* (C57BL/6 mouse alopecia model); Clinical RCT (postmenopausal women, double-blind)	Oral 200–400 mg/kg or topical 1–2 mg/day; MAC 50–100 μM ginsenosides; 12–24 weeks	Positive: minoxidil 2%; Negative: saline/vehicle	Prolonged anagen by 25%–35%; increased follicle proliferation by 20%–40% and hair density by 15%–30% via anti-apoptotic and VEGF upregulation	Limited menopausal-specific RCTs (mostly general alopecia); underrepresentation of non-Asian ethnicities (<20%); no long-term safety data (>1 year); potential bias from small n (50–100); high doses in some animal studies reduce translatability	Zaid et al., 2017; Choi (2018), Iwabuchi et al. (2024)
*Aloe vera* (L.) Burm.f. [Asphodelaceae; Aloes folii succus]	Aqueous gel extract, standardized to 20%–30% polysaccharides/enzymes (HPLC: acemannan, aloin)	Clinical (small open-label trials, mixed alopecia); *In vitro* (human dermal papilla cells)	Topical 10%–20% gel/mask; MAC 5%–20%; 8–12 weeks	Positive: minoxidil 2%; Negative: vehicle	Modest hair density increase 10%–25%; follicle proliferation 20%–30% via antioxidative and hydration mechanisms	No menopausal-specific RCTs found; small samples (n = 15–50); short durations; potential placebo/publication biases; lacks hormonal context in models (overestimation in non-menopausal); no systemic/long-term safety data in diverse cohorts	Choi et al. (2024), Nandi et al. (2025)
*Serenoa repens* (W.Bartram) Small [Arecaceae; Serenoae repentis fructus]	Liposterolic extract, standardized to 85%–95% fatty acids/sterols (HPLC: lauric acid, β-sitosterol)	Clinical RCT (mixed gender alopecia); *In vivo* (rat androgen model)	Oral 200–320 mg/day; MAC 10–50 μM; 6–24 months	Positive: finasteride 1 mg; Negative: placebo	DHT reduction 30%–60%; hair loss arrest/improvement in 60% vs. 11% placebo; inhibits 5α-reductase	Limited menopausal-specific evidence (mostly male AGA); small n (50–100); mixed gender trials reduce applicability; mild side effects (GI, libido); no head-to-head vs. HRT; ethnic underrepresentation	Evron et al. (2020), Nguyen et al. (2025)
*Salvia rosmarinus* Spenn. [Lamiaceae; Rosmarini folium]	Essential oil extract, standardized to 10%–20% carnosic acid/rosmarinic acid (HPLC)	*In vivo* (C57BL/6 mouse telogen model); Clinical RCT (androgenetic alopecia)	Topical 2–5 mg/day or 3%–5% oil; MAC 5–15 μM; 6–12 weeks	Positive: minoxidil 2%; Negative: vehicle	Improved hair regrowth; increased density 20%–30%; promotes microcirculation and inhibits 5α-reductase	Few menopausal-specific studies; short durations; potential skin irritation; models lack hormonal simulation (limited relevance to menopause); small n (50–100); overestimation in non-menopausal alopecia	Panahi et al. (2015), Begum et al. (2023)
*Eclipta prostrata* (L.) L. [Asteraceae; Ecliptae herba] (syn. *Eclipta alba* (L.) Hassk.)	Methanolic/herbal oil extract, standardized to 15%–25% wedelolactone/coumarins (HPLC)	*In vitro* (dermal papilla/keratinocyte cells); *In vivo* (albino rat anagen induction)	Topical 1–2 mg/day; MAC 20–50 μM; 8–12 weeks	Positive: minoxidil; Negative: vehicle	Increased proliferation 25%–35%; anagen induction; hair growth promotion via antioxidant/anti-apoptotic effects	Limited RCTs (mostly preclinical); no menopausal-specific trials; small animal models; potential hepatotoxicity risks; gaps in diverse cohorts and long-term data	Kumari et al. (2021), Susantha Priyadarshani et al. (2023)
*Reynoutria multiflora* (Thunb.) Moldenke [Polygonaceae; Polygoni multiflori radix] (syn. *Polygonum multiflorum* Thunb.)	Ethanolic root extract, standardized to 20%–30% stilbenes/emodin (HPLC: 2,3,5,4′-tetrahydroxystilbene glucoside)	*In vivo* (C57BL/6 mouse stress-induced alopecia); *In vitro* (follicle cells)	Oral/topical 100–300 mg/kg; MAC 10–50 μM; 8–16 weeks	Positive: minoxidil; Negative: saline	Elongated anagen 20%–30%; delayed catagen; hair growth promotion via Shh/β-catenin upregulation	Limited human RCTs (mostly animal/*in vitro*); hepatotoxicity concerns at high doses; no menopausal-specific data; small samples; translatability issues from stress models	Shin et al. (2020), Qian et al. (2024)
*Urtica dioica* L. [Urticaceae; Urticae radix]	Aqueous/ethanol root extract, standardized to 10%–20% lignans/sterols (HPLC: β-sitosterol)	*In vitro* (hair matrix keratinocytes); *Ex vivo* (follicle organ culture)	Topical 1%–5%; MAC 20–100 μM; 4–8 weeks	Positive: finasteride; Negative: DMSO	Promoted proliferation; DHT inhibition; hair growth stimulation via anti-inflammatory effects	Sparse RCTs; no menopausal-specific studies; mostly *in vitro*/*ex vivo*; potential allergies; limited dose optimization; ethnic gaps	Pekmezci et al. (2018), Pekmezci and Türkoğlu (2023)
*Vitellaria paradoxa* C.F.Gaertn. [Sapotaceae; Butyrospermi parkii butyrum]	Butter extract, standardized to 40%–60% oleic acid/triterpenes (HPLC)	Observational/clinical (small trials, general hair care)	Topical 10%–20%; Duration 8–12 weeks	Positive: commercial moisturizers; Negative: vehicle	Improved scalp hydration; reduced inflammation; modest hair strengthening	Limited evidence; no menopausal-specific RCTs or *in vivo*/*in vitro* studies 2015-2025; small n; gaps in mechanisms/dose; potential bias from non-blinded trials	Ezekwe et al. (2020), Ayanlowo et al. (2021)
*Prunus africana* (Hook.f.) Kalkman [Rosaceae; Pruni africanae cortex]	Bark extract, standardized to 13%–14% β-sitosterol (HPLC)	*In vitro* (prostate cells, extrapolated to hair); Small clinical (BPH-related)	Oral 100–200 mg/day; MAC 50–100 μM; 3–6 months	Positive: finasteride; Negative: placebo	DHT reduction 30%–40%; anti-inflammatory; potential hair benefits via androgen modulation	No direct menopausal hair loss studies; mostly prostate-focused; limitations in female cohorts; side effects (GI); gaps in hair-specific RCTs	Rubegeta et al. (2022)
*Simmondsia chinensis* (Link) C.K.Schneid. [Simmondsiaceae; Simmondsiae chinensis semen]	Oil extract, standardized to 40%–50% wax esters (HPLC)	*In vitro* (scalp barrier models); Clinical (general conditioning)	Topical 5%–10%; Duration 4–8 weeks	Positive: mineral oil; Negative: vehicle	Balanced sebum; prevented breakage; hydration support	Limited hair loss evidence; no menopausal RCTs 2015-2025; mostly cosmetic studies; gaps in mechanisms for alopecia; small samples	Gad et al. (2021)

### 4.1 Asia: traditional chinese medicine and ayurveda

In Asia, Traditional Chinese Medicine (TCM) and Ayurveda use botanical remedies like ginseng and bhringraj to promote hair vitality, traditionally viewed as ways to balance vital energies such as qi or doshas to support women’s holistic health during menopause (Shin et al., 2020; Kesika et al., 2023). *Panax ginseng* C.A.Mey. [Araliaceae; Ginseng radix et rhizoma] invigorates qi and enhances follicle proliferation via ginsenosides. *Eclipta alba* (L.) Hassk. [Asteraceae; Ecliptae herba], or bhringraj, aids hair darkening and growth through compounds like wedelolactone. *Polygonum multiflorum* Thunb. [Polygonaceae; Polygoni multiflori radix], commonly known as He Shou Wu, focuses on anti-aging and anagen extension with stilbenes and anthraquinones.

These remedies address hormonal imbalances and oxidative stress, with preclinical and clinical evidence showing improvements in hair density and cycle regulation. However, research often lacks menopausal-specific focus and ethnic diversity. For detailed extract compositions, study models, doses, findings, and gaps (e.g., hepatotoxicity concerns), refer to Table 2 (Choi, 2018; Han et al., 2015; Lin et al., 2015; Iwabuchi et al., 2024). By revitalizing these traditions, Asian ethnopharmacology fosters cultural resilience and autonomy for women in resource-limited settings.

### 4.2 Europe and mediterranean: botanical drug infusions and oil

European and Mediterranean ethnopharmacology draws from ancient Greek, Roman, and folk traditions, emphasizing aromatic infusions and oils for scalp health and hair restoration that often improve circulation and reduce androgen effects (Zaid et al., 2017; Bin Rubaian et al., 2024; Leonti and Verpoorte, 2017). *Salvia rosmarinus* Spenn. [Lamiaceae; Rosmarini folium], or rosemary, promotes microcirculation with carnosic and rosmarinic acids, mimicking minoxidil’s effects. Combined with lavender (*Lavandula angustifolia* Mill.), it reduces inflammation and stress. Nettle root (*Urtica dioica* L.) acts as a DHT blocker. Horsetail (*Equisetum arvense* L.), ylang-ylang (*Cananga odorata* (Lam.) Hook. f. and Thomson), and peppermint (*Mentha × piperita* L.) provide silica and circulatory benefits.

These remedies are typically applied as topical blends or infusions, showing promise in enhancing hair density and soothing scalps in RCTs. However, studies are limited by short durations and lack of hormonal context. For specifics on extracts, trials, efficacy metrics, and limitations (e.g., ethnic underrepresentation), see Table 2 (Panahi et al., 2015; Smith et al., 2021; Greger and Landberg, 2024; Patel et al., 2025). These sensory empower women through self-care practices, bridging ancient wisdom with modern needs in diverse European contexts.

### 4.3 Americas: indigenous and native plant extracts

In the Americas, Indigenous and Latin American traditions use native plants like saw palmetto, aloe vera, and jojoba to counter hormonal hair loss, emphasizing harmony with nature and community knowledge (Celidwen et al., 2023; Thimmannagari et al., 2025). *Serenoa repens* (W.Bartram) Small [Arecaceae], or saw palmetto, inhibits 5α-reductase to reduce DHT. *Aloe vera* (L.) Burm. f. [Asphodelaceae] hydrates and nourishes via polysaccharides and vitamins. Jojoba (*Simmondsia chinensis* (Link) C.K.Schneid.) [Simmondsiaceae] mimics sebum for scalp balance.

Evidence from RCTs and ethnobotanical surveys supports improvements in alopecia and effluvium, especially in postmenopausal women. Gaps include small sample sizes and limited long-term safety data. For detailed pharmacological profiles, doses, study outcomes, and challenges, refer to Table 2 (Evron et al., 2020; Catalano et al., 2024). These remedies promote women’s empowerment by preserving Indigenous practices and addressing health inequities in underserved communities.

### 4.4 Africa and other regions: nutrient-rich butters and extracts

African ethnopharmacological traditions span diverse ecosystems from sub-Saharan regions to North Africa, utilizing indigenous plants like shea butter, moringa, and African cherry to address hair vitality. These remedies are often incorporated into communal rituals that emphasize harmony with nature and women’s central roles in health preservation (Rubegeta et al., 2022; von Maltitz and Bahta, 2024; Degbe et al., 2025). *Vitellaria paradoxa* C.F.Gaertn. [Sapotaceae; Butyrospermi parkii butyrum], commonly known as shea butter, nourishes and protects the scalp with fatty acids, delivering anti-inflammatory effects. *Moringa oleifera* Lam. [Moringaceae; Moringae folium] provides antioxidants, anti-alopecia properties, and DHT inhibition through flavonoids, vitamins, and isothiocyanates. *Prunus africana* (Hook.f.) Kalkman [Rosaceae; Pruni africanae cortex], or pygeum, reduces inflammation via beta-sitosterol. Additional examples include pumpkin seed (*Cucurbita pepo* L.) for DHT inhibition and anagen promotion, olive oil (*Olea europaea* L.) for anagen stimulation via Wnt/β-catenin signaling, and black seed (*Nigella sativa* L.) for antioxidant and hair growth promotion through thymoquinone.

These remedies, typically applied as topical butters, extracts, or infusions, demonstrate promise in improving alopecia and telogen effluvium in RCTs and ethnobotanical surveys, particularly among postmenopausal women. However, evidence is constrained by small sample sizes, short study durations, and insufficient long-term safety data. For detailed extract compositions, study models, doses, efficacy metrics (e.g., hair density improvements), and limitations (e.g., underreporting in low-resource settings), refer to Table 2 (Pareek et al., 2023; Megnanou and Niamke, 2015; Isaac et al., 2023; Camilleri and Blundell, 2024). By revitalizing these traditions, African ethnopharmacology fosters cultural resilience and autonomy for women, addressing health inequities in underserved communities.

## 5 Mechanisms, evidence, and safety profiles

### 5.1 Multi-target mechanisms of action

Botanical drugs address menopausal hair loss through synergistic, multi-target mechanisms. Phytoestrogens, such as soy isoflavones, mimic estrogen to extend the anagen phase and mitigate follicular miniaturization. Anti-androgens, including fatty acids in saw palmetto, inhibit 5α-reductase, reducing DHT levels by up to 40% in androgen-sensitive regions. Antioxidants like ginsenosides in ginseng and carnosic acid in rosemary counteract oxidative stress and inflammation, improving microcirculation and nutrient delivery to prolong follicle viability (Rinaldi et al., 2023; Ge et al., 2019).

Preclinical models, including *in vitro* dermal papilla assays and mouse alopecia simulations, elucidate these pathways. However, they frequently lack a comprehensive hormonal context, such as estrogen decline, limiting their direct applicability to menopause. Key gaps include overreliance on general alopecia models without menopausal specificity.

### 5.2 Efficacy evidence and quality assessment

Among the 78 included studies, efficacy varies. For instance, ginseng RCTs (n = 80–100 postmenopausal women, double-blind) demonstrate 20%–40% follicle proliferation via ginsenosides (Choi, 2018). Saw palmetto clinical trials (n = 50–100, mixed gender) report 20%–35% density improvements through DHT inhibition (Evron et al., 2020). Rosemary oil RCTs (n = 100) increase density by up to 27% via circulation enhancement (Panahi et al., 2015). *In vitro* and animal data, such as aloe vera increasing proliferation by 25% in dermal papilla cells, support these mechanisms, though they may overestimate translatability due to absent hormonal simulation.

Overall, most evidence comes from *in vitro* or animal studies (70% of studies), with only 30% from clinical trials, often not specific to menopausal women. A notable limitation in some animal models is the use of high doses (>1 g/kg), reducing relevance to human applications. In this review, standalone *in silico* and network studies without experimental validation were deliberately excluded to prioritize more robust and reproducible methods (Kim et al., 2022). Refer to Table 2 for comprehensive study summaries, compositions, and assessments.

### 5.3 Safety profiles and regulatory considerations

Botanical drugs generally exhibit low toxicity, with mild gastrointestinal effects occurring with ginseng (<5% incidence in RCTs) and rare scalp irritation arising from rosemary or saw palmetto (Bin Rubaian et al., 2024). No severe adverse events have been reported in menopausal cohorts. However, hepatotoxicity risks with high-dose *Polygonum multiflorum* necessitate caution.

Standardization through HPLC for key markers (e.g., ginsenosides or fatty acids) is essential. Pharmacovigilance via VigiBase monitors rare interactions, such as ginseng with antihypertensives. Regulatory frameworks vary, with EMA herbal monographs contrasting FDA supplements, underscoring the need for unified global standards and Nagoya-compliant sourcing to ensure equity (Thakkar et al., 2020).

## 6 Challenges, future directions, and conclusion

This mini-review emphasizes critical research challenges and future priorities rather than exhaustive coverage. The field of ethnopharmacology for menopausal hair loss remains fragmented, requiring focused efforts to advance equitable solutions.

### 6.1 Research challenges

Key gaps include the scarcity of menopausal-specific RCTs (∼25% of 78 studies), often featuring small samples (n < 100) and short durations (<12 weeks). Studies overly rely on irrelevant models, such as general *in vitro* assays without estrogen-androgen simulation, and include unsubstantiated claims of multi-target effects without dose-response data or proper controls.

For instance, many report anti-inflammatory activity based on *in vitro* cytokine reduction but fail to evaluate minimal active concentrations or relevance to scalp microcirculation in postmenopausal women. Standardization issues persist, with extracts varying in metabolite content (e.g., 20%–40% ginsenosides in ginseng), leading to inconsistent efficacy. Ethnic underrepresentation and lack of long-term safety data (>12 months) exacerbate disparities, particularly in non-Asian cohorts where traditional uses may not align with modern pharmacology.

### 6.2 Future research needs and priorities

To address these gaps, prioritize large-scale, menopausal-specific RCTs (n > 200, >12 months) with diverse ethnic cohorts to validate efficacy beyond small pilots. Develop standardized extraction protocols compliant with pharmacopoeia standards, including MAC reporting, for reproducibility.

Pursue interdisciplinary trials integrating ethnobotany (e.g., cultural validation of traditional uses) with modern pharmacology (e.g., *in vivo* models simulating estrogen decline). Enhance model relevance by developing *ex vivo* menopausal scalp models over generic *in vitro* assays. Conduct equitable, community-engaged studies in low-resource settings to bridge socioeconomic divides and adhere to Nagoya Protocol ethics.

### 6.3 Limitations

This mini-review synthesizes key ethnopharmacological evidence on botanical drugs for menopausal hair loss. However, limitations exist. First, it is not exhaustive; the vast scope of global traditional medicines necessitated a focus on representative examples from select regions, potentially overlooking lesser-documented remedies or emerging studies from underrepresented areas like Oceania or the Middle East.

Second, study heterogeneity precludes formal meta-analysis, with sources ranging from preclinical models to small-scale RCTs, introducing potential bias in narrative synthesis (e.g., overreliance on *in vitro* data for mechanisms like anti-inflammation that may not translate to menopausal contexts).

Third, reliance on English-language publications and databases like PubMed may exclude valuable non-English ethnobotanical sources, contributing to cultural and geographic disparities. Many 2025 citations are recent or ahead-of-print, limiting long-term validation.

Future reviews should incorporate multilingual searches and systematic meta-analyses to enhance comprehensiveness and equity in women’s health research.

In conclusion, ethnopharmacological botanical drugs empower women by offering culturally resonant, sustainable solutions for menopausal hair loss, transforming this phase into one of resilience and autonomy.
